# The Effect of Belief in Free Will on Prejudice

**DOI:** 10.1371/journal.pone.0091572

**Published:** 2014-03-12

**Authors:** Xian Zhao, Li Liu, Xiao-xiao Zhang, Jia-xin Shi, Zhen-wei Huang

**Affiliations:** 1 School of Psychology, Beijing Normal University, Beijing, China; 2 Department of Psychology, University of Kansas, Lawrence, Kansas, United States of America; Saarland University, Germany

## Abstract

The current research examined the role of the belief in free will on prejudice across Han Chinese and white samples. Belief in free will refers to the extent to which people believe human beings truly have free will. In Study 1, the beliefs of Han Chinese people in free will were measured, and their social distances from the Tibetan Chinese were used as an index of ethnic prejudice. The results showed that the more that Han Chinese endorsed the belief in free will, the less that they showed prejudice against the Tibetan Chinese. In Study 2, the belief of the Han Chinese in free will was manipulated, and their explicit feelings towards the Uyghur Chinese were used as an indicator of ethnic prejudice. The results showed that the participants in the condition of belief in free will reported less prejudice towards Uyghur Chinese compared to their counterparts in the condition of disbelief in free will. In Study 3, white peoples’ belief in free will was manipulated, and their pro-black attitudes were measured as an indirect indicator of racial prejudice. The results showed that, compared to the condition of disbelief in free will, the participants who were primed by a belief in free will reported stronger pro-black attitudes. These three studies suggest that endorsement of the belief in free will can lead to decreased ethnic/racial prejudice compared to denial of the belief in free will. The theoretical and practical implications are discussed.

## Introduction

Recent worldwide intergroup antagonisms, including the increasing controversies due to the self-immolations of Tibetans in protest against the Han Chinese that have occurred since 2009, the Trayvon Martin shooting in the United States in 2012 and the Marikana massacre in South Africa in 2012, have appalled the public. These tragedies confront us with two pressing questions: Do people believe that human beings truly have free will to control their behaviors? Moreover, is there an alternative way to reduce ethnic/racial prejudice beyond our existing knowledge? An interesting research question emerges from the combination of these two issues; can the endorsement of a belief in free will lead to less ethnic/racial prejudice? This question is not arbitrary. In the present research, we performed three studies to address this question.

### Belief in Free Will

Belief in free will refers to the belief that human beings are autonomous agents that can determine their choices and behaviors from multiple options [1]. In contrast, disbelievers in free will believe that human beings’ actions are merely illusions that are essentially determined by the principles of the universe [2]. When studying the belief in free will, researchers usually include a manipulation of disbelief in free will as a comparison. Living in a social world, human beings may face multiple choices in certain situations. Some choices are naturally impulsive, and some choices are socially desirable but may conflict with the natural responses. As a form of action control, free will is a type of mental energy that can restrain and control the natural responses and promote rational behaviors that are more harmless or more beneficial to the ingroup [2]–[3]. Therefore, when people believe in free will, they may exert greater levels of self-control. It has been found that undermining individuals’ free will leads to reduced intentional inhibitions [4]–[5], and when priming willpower is unlimited, the effect of ego-depletion (self-control is low after the prior exertion of self-control) is ameliorated [6]–[7].

In line with the above findings, stronger beliefs in free will predict and facilitate a series of positive outcomes that require higher levels of self-control, for example, better job performance [8], and learning from emotional experiences [9]. Individuals who have been primed with free will are more likely to write first-person narratives related to higher levels of self-control that include factors such as achieving goals and moral behaviors [3]. However, disbelief in free will causally leads to a series of negative outcomes that produced by lower levels of self-control, for example, more aggression towards others, hesitance in pro-social helpfulness [10], and increased cheating behavior on tests [11].

### Belief in Free Will and Prejudice

We hypothesize that the belief in free will can predict and lead to decreased prejudice compared to disbelief in free will. This hypothesis is based on the following two rationales. First, as stated above, free will is a form of action control that serves as a type of volitional willpower. Therefore, when participants are primed with free will, self-control may be correspondingly elevated. Second, previous studies have found that poor self-control is correlated with, and causally leads to, prejudice against outgroups [12]–[14]. When self-control is bolstered by drinking glucose water, prejudice is reduced relative to control conditions [15].

Thus, it is reasonable to make the following predictions: 1) greater beliefs in free will is associated with decreased prejudice against outgroup members (hypothesis 1); and 2) the belief in free will leads to decreased prejudice against outgroup members compared to disbelief in free will (hypothesis 2).

Notably, the concept of free will has been widely debated in both philosophy and psychology [16]. The above predictions are based on Baumeister’s (2008) notion that a belief in free will involves more stringent self-regulation. However, as noted by Carey and Paulhus (2013), the reverse predictions are certainly tenable when understanding belief in free will as personal responsibility for behaviors. According to Weiner’s (1993) attribution model [17], a belief in free will may also encourage people to be critical of others’ misbehavior [18]. Specifically, greater levels of belief in free will may motivate people to make more controllable attributions to outgroups’ negative attributes, blame them for their misbehaviors, and thus lead to increased prejudice [17], [19], which forms the opposite hypotheses to our existing ones. However, the attribution process may not occur in the current design. As indicated by Weiner [20]–[21], specific behaviors, events or scenarios are essential for engaging in either reactive or spontaneous attributions. We thus infer that an attribution process may not be readily activated by the general attitude measures or priming techniques as used in the current research. So this interesting topic is out of scope of the current research. The focus of the current research is the role of belief in free will on prejudice against outgroups from one’s own perspective.

### The Current Research

The current research was conducted in two different socio-cultural backgrounds: China and the Western world. These environments each have distinct issues that are related to prejudice, and addressing all of these issues is important. China is a multi-ethnic country in which the Han Chinese compose the national majority, and the Tibetan Chinese and the Uyghur Chinese are the two main ethnic minorities. The tensions between the ethnic minorities (especially the Tibetan and Uyghur Chinese) and the Han majority are always present due to historical reasons and contemporary incidents. Both the Tibetan Chinese and Uyghur Chinese are usually negatively stereotyped as violent, aggressive, and rustic by the Han Chinese [22]. In many Western countries, blacks are usually negatively stereotyped as uneducated, unintelligent, and violent [23], and blacks are the subjects of either blatant or subtle prejudice by the white majority (e.g., in the United States and Britain) due to the history of slavery or other political and economic reasons.

In the current research, we employed different participant samples (Han Chinese in Studies 1 and 2, and whites in Study 3) and different methods to test our hypotheses. Study 1 aimed to test hypothesis 1. In this study, the beliefs of Han Chinese participants regarding free will were measured, and their social distances from the Tibetan Chinese were used as indices of ethnic prejudice. Studies 2 and 3 aimed to test hypothesis 2. In Study 2, the beliefs of the Han Chinese in free will were manipulated, and their explicit feeling towards Uyghur Chinese was used as an indicator of ethnic prejudice. In Study 3, the beliefs of whites were manipulated, and their pro-black attitudes were measured as an indirect indicator of racial prejudice.

## Study 1

This study aimed to test hypothesis 1: greater belief in free will is associated with lower prejudice against outgroup members. Specifically, we predicted that there would be a negative correlation between the belief of the Han Chinese in free will and their social distance from the Tibetan Chinese. Both variables were measured via questionnaires.

### Method

#### Ethics Statement

This study was reviewed and approved by the Committee of Protection of Subjects at Beijing Normal University. All participants provided written informed consent before the study, and they were fully debriefed at the end of the research according to the established guidelines of the committee. This procedure was followed in Studies 2 and 3 as well.

#### Participants

The participants were 70 college students (12 males and 58 females) in Beijing who self-identified as Han Chinese. They were recruited by the first author through face-to-face encounters at the library of Beijing Normal University. The participants were informed that the study sought to investigate general social beliefs. The participants completed the questionnaires individually. The average age of the participants was 27.7 years (*SD* = 4.63).

#### Measures

*Belief in Free Will Measure.* Belief in free will was measured via 6 items (Cronbach’s α = 0.75) in Chinese that were translated from the Free Will and Determinism Scale (FAD-Plus) [24]. For example, one item was “Strength of mind can always overcome the body’s desires” (Questionnaire S1). The item “People have complete free will” on the Free Will and Determinism Scale was dropped in this research because the term “complete free will” was impenetrable to those who without special knowledge. Therefore, only the remaining six items were used. The participants were asked to choose a number from 1 to 7 to indicate their level of agreement with each item. The average score of each of these 6 items was calculated as the index of belief in free will; higher scores represented stronger beliefs in free will.

*Social Distance Measure.* A 5-item version of the Bogardus Social Distance Scale [25] was used to measure the participants’ prejudice against Tibetan Chinese (Cronbach’s α = 0.79). For example, one sample item was “I do not mind living in the same community with Tibetans” (Questionnaire S2). The participants were instructed to indicate the extent to which they endorsed each statement on 7-point Likert scales (1 =  strongly disagree; 7  =  strongly agree). The average score of the five items was calculated as an indicator of prejudice, and higher scores indicated stronger prejudices.

#### Procedure

The participants were instructed to complete several questionnaires that included belief in free will, social distance measures, and other measures that were unrelated to the current study. After the study, each participant was given a small gift as a token of gratitude for the time and effort of their participation.

### Results and Discussion

**Figure 1:**
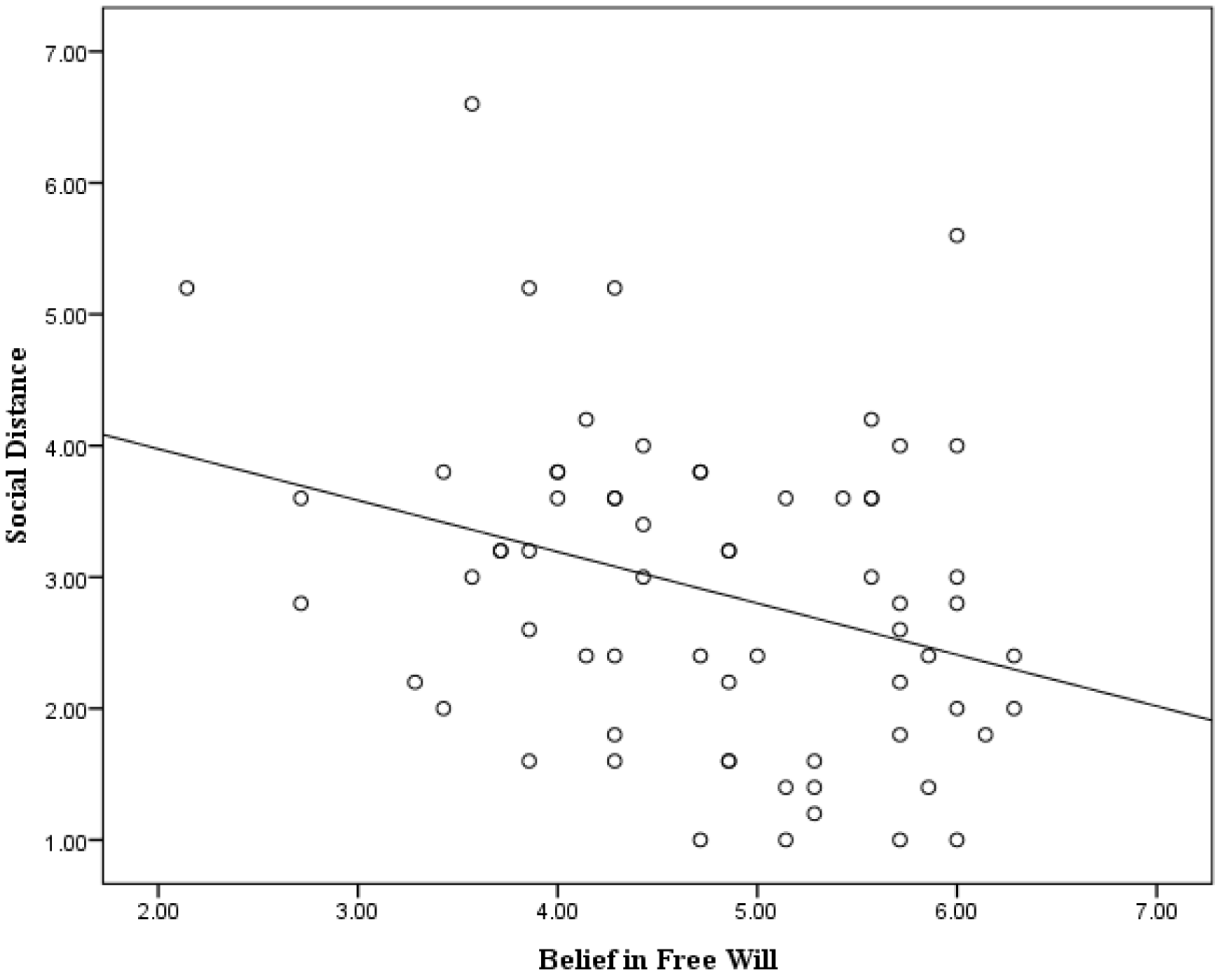
Scatter plot with regression line between the belief in free will of the Han Chinese participants and their social distance from the Tibetan Chinese.

The responses of four participants regarding their beliefs in free will were missing (5.7%); however, all participants’ responses about their beliefs in free will and their social distances were within 3 standard deviations of the mean. The mean score for belief in free will was 4.77 (*SD*  =  0.97), and the mean social distance score was 2.90 (*SD*  =  1.19). These results revealed a significant negative correlation between belief in free will and prejudice against the Tibetan Chinese (*r* = –0.316, *p* = .010). A regression analysis confirmed our prediction that greater Han Chinese beliefs in free would significantly predict less prejudice against Tibetan Chinese (*Beta*  =  –0.316, *t*(65)  = –2.67, *p*  = .010, *R^2^* = 0.10). Figure 1 illustrates the relevant scatter plot and regression line. Thus, hypothesis 1 was confirmed by our results.

However, there were two limitations to this study. First, this study employed a cross-sectional design, which makes it difficult to infer the causality. Second, the target of prejudice was limited to Tibetan Chinese, and thus it is not clear whether the pattern of the relationship can be generalized to other ethnic groups in China. To overcome these limitations, we used a priming technique in Study 2 to manipulate belief and disbelief in free will, and we tested the causal link between the beliefs of the Han Chinese in free will and their prejudices against another ethnic outgroup, the Uyghur Chinese.

## Study 2

This study aimed to test the hypothesis 2, which was a follows: belief in free will leads to reduced prejudice against outgroup members compared to disbelief in free will. Specifically, we predicted that the Han Chinese participants primed with a belief in free will would display more positive explicit feelings towards the Uygur Chinese than would their counterparts who had been primed with disbelief in free will.

### Method

#### Participants

A total of 34 college students (10 males and 24 females) from Beijing who self-identified as Han Chinese participated in this study. The participants were recruited for this study via flyers that were posted on an Internet forum of Beijing Normal University. The participants were informed that this study would investigate social attitudes. The average age of the participants was 21.6 years (*SD* = 2.65). The participants were assigned to belief in free will (*N* = 17) or disbelief in free will conditions (*N* = 17) via a simple randomization based on sequential order (one participant was assigned into one condition, and next participant was assigned into the opposite condition).

#### Materials

*Priming for Belief in Free Will.* As adapted from Vohs and Schooler’s paradigm [11], to prime beliefs in free will, the participants in the belief in free will condition were instructed to complete two tasks. In the first task, the participants were asked to use their own words to summarize the main ideas of the six statements that were selected from the belief in free will subscale of the Free Will and Determinism Scale [24] that were used in Study 1. For example, one sample item was “People have complete control over the decisions they make”. In the second task, the participants were asked to recall their own experiences that would support the main ideas of the six statements. The participants completed the tasks on computers. In the disbelief in free will condition, the participants were instructed to complete two similar tasks with the exceptions that the six statements were different. Specifically, five statements were selected from the fatalistic determinism subscale of the Free Will and Determinism Scale [24]. For example, one sample statement was “The future has already been determined by fate”. The remaining statement was “Fate determines one’s success and failure” (Priming materials S1).

*Feeling Thermometer*. A feeling thermometer based on that of Dasgupta and Greenwald [26] was used to assess the favorability/unfavorability of the participants’ explicit feelings towards Uyghur Chinese. The participants were instructed to indicate their attitudes towards Uyghur Chinese on a scale from 1 (extremely cold) to 100 (extremely warm).

#### Procedure

The participants were instructed to complete the priming task and the feeling measurement as two separate studies in two distinct rooms. Thus, the participants were blinded to the true purpose of our study. After the study, each participant was paid RMB¥10 for their participation.

### Results and Discussion

**Figure 2:**
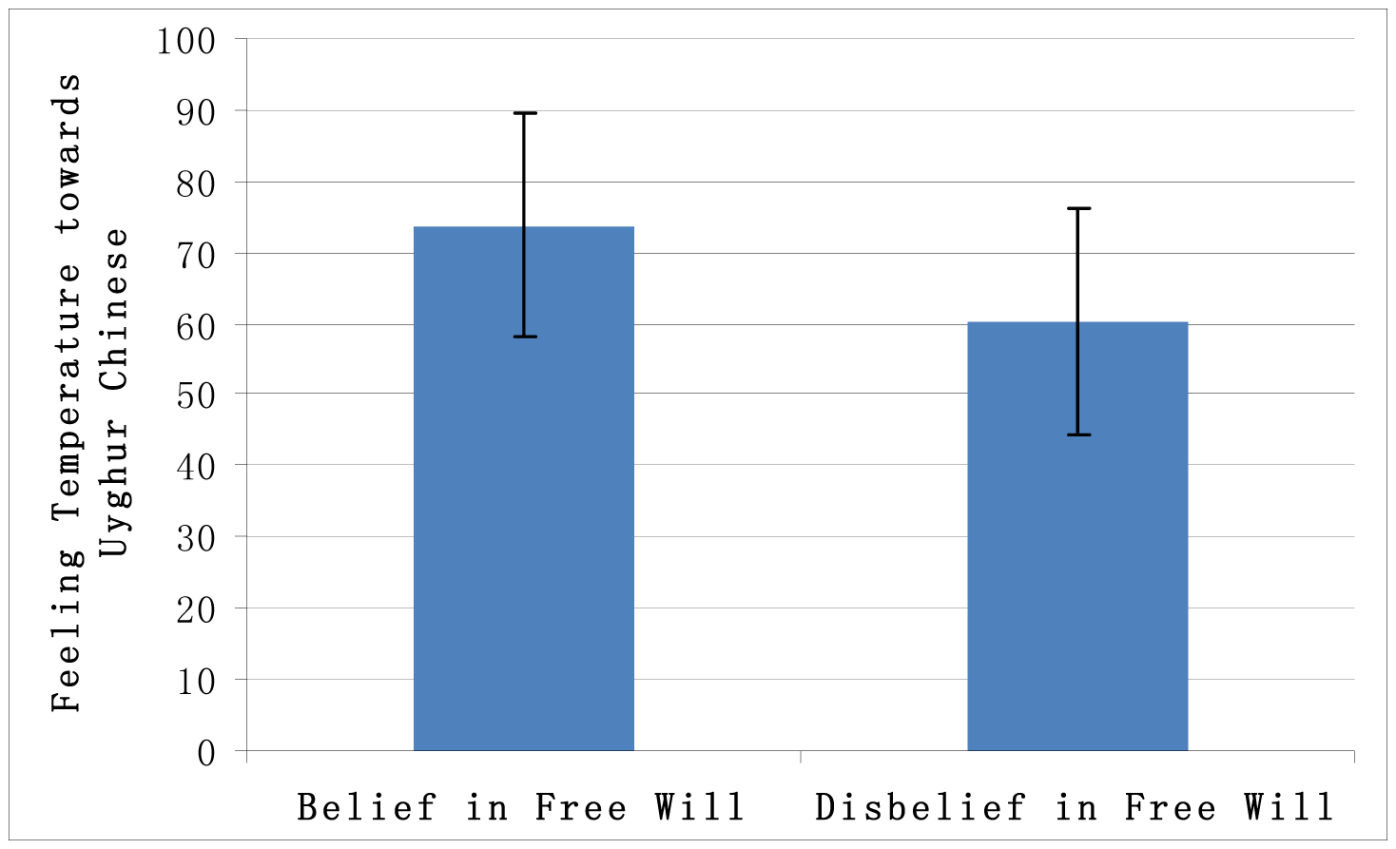
Feeling temperatures towards the Uyghur Chinese under the conditions of belief in free will and disbelief in free will.

All participants’ responses about their feeling temperatures were within 3 standard deviations of the mean; thus, all responses were included in the subsequent analyses. We computed independent t-tests to examine the effects of priming for a belief in free will and priming for a disbelief in free will on prejudice against Uyghur Chinese. The results showed that the participants in the condition of belief in free will reported significantly warmer temperatures towards Uyghur Chinese (*M* = 73.82, *SD* = 15.57) compared to the participants in the disbelief in free will condition (*M* = 60.29, *SD* = 16.05), *t*(32)  = –2.50, *p* = 0.018, *Cohen’s d* = 0.86 (Figure 2). Thus, these results confirmed hypothesis 2.

One limitation of Studies 1 and 2 is that the targets of prejudice were both Chinese ethnic minorities. Thus, whether our results are applicable to Western cultural contexts remains to be determined. Next, to further test the cross-cultural validity of the current findings, in Study 3, we tested hypothesis 2 in a sample of white people and included blacks as the target of prejudice.

## Study 3

This study aimed to further test hypothesis 2: the belief in free will leads to reduced prejudice against outgroup members compared to disbelief in free will. Specifically, we predicted that the white participants who were primed with a belief in free will would exhibit greater pro-black attitudes than would their counterparts that had been primed with a disbelief in free will.

### Method

#### Participants

In total, 63 participants (34 males and 29 females) who self-reported as white participated in this study. The participants’ ages ranged from 17 to 67 (*M* = 39.6, *SD* = 15.0). They were from South Africa (30.3%), Britain (24.2%), the United States (9.1%), and other European countries. The data were collected at the airports of Cape Town, Dubai and Beijing and on two flights between these airports during the journey of the first author who was attending the 30^th^ International Congress of Psychology. Each participant was individually invited to take part in this study. The participants were blinded to the true purpose of this study. The participants were informed that this study sought to investigate reading and social beliefs in general. The participants were randomly assigned to one of two priming conditions according to a coin flip: the belief in free will condition (*n* = 35), and the disbelief in free will condition (*n* = 28).

#### Materials

*Priming of Belief in Free Will*. The participants were instructed to read a passage that was fabricated to be a paragraph from *Science Magazine.* The contents of the passage of each condition were revised from No et al. [27]. In the condition of the belief in free will, the passage supported the usefulness of volitional control for humankind and advocated the position that human behaviors are determined by our intentions and desires. In the condition of disbelief in free will, the passage supported the uselessness of volitional control for humankind and advocated the position that many human behaviors are not determined by our intentions and desires (Priming materials S2).

To verify the efficacies of these manipulations, after reading the passage, the participants were asked to underline a sentence that represented the main idea of the paragraph and to select one option that correctly summarized the paragraph.

*Pro-Black Attitude Measure*. Six items from the Pro-Black Attitudes Questionnaire [28] were used to assess the participants’ prejudices against black people (*Cronbach’s* α = 0.60). For example, one item was “Most blacks are no longer discriminated against” (Questionnaire S3). The participants were instructed to indicate the extent to which they endorsed each statement on 5-point Likert scales (1  =  strongly disagree and 5  =  strongly agree). Higher scores represented more favorable attitudes towards blacks.

#### Procedure

The participants were instructed to read the priming material regarding beliefs in free will (or disbeliefs in free will) and then complete a pro-black attitudes measure, in that order.

### Results and Discussion

All participants’ responses on the pro-black scales were within 3 standard deviations of the mean. The manipulation check revealed that all of the participants correctly underlined the sentence that corresponded to the main idea of the paragraph and selected the option that correctly summarized the paragraph. Thus, all of the participants were included in the subsequent analyses.

**Figure 3:**
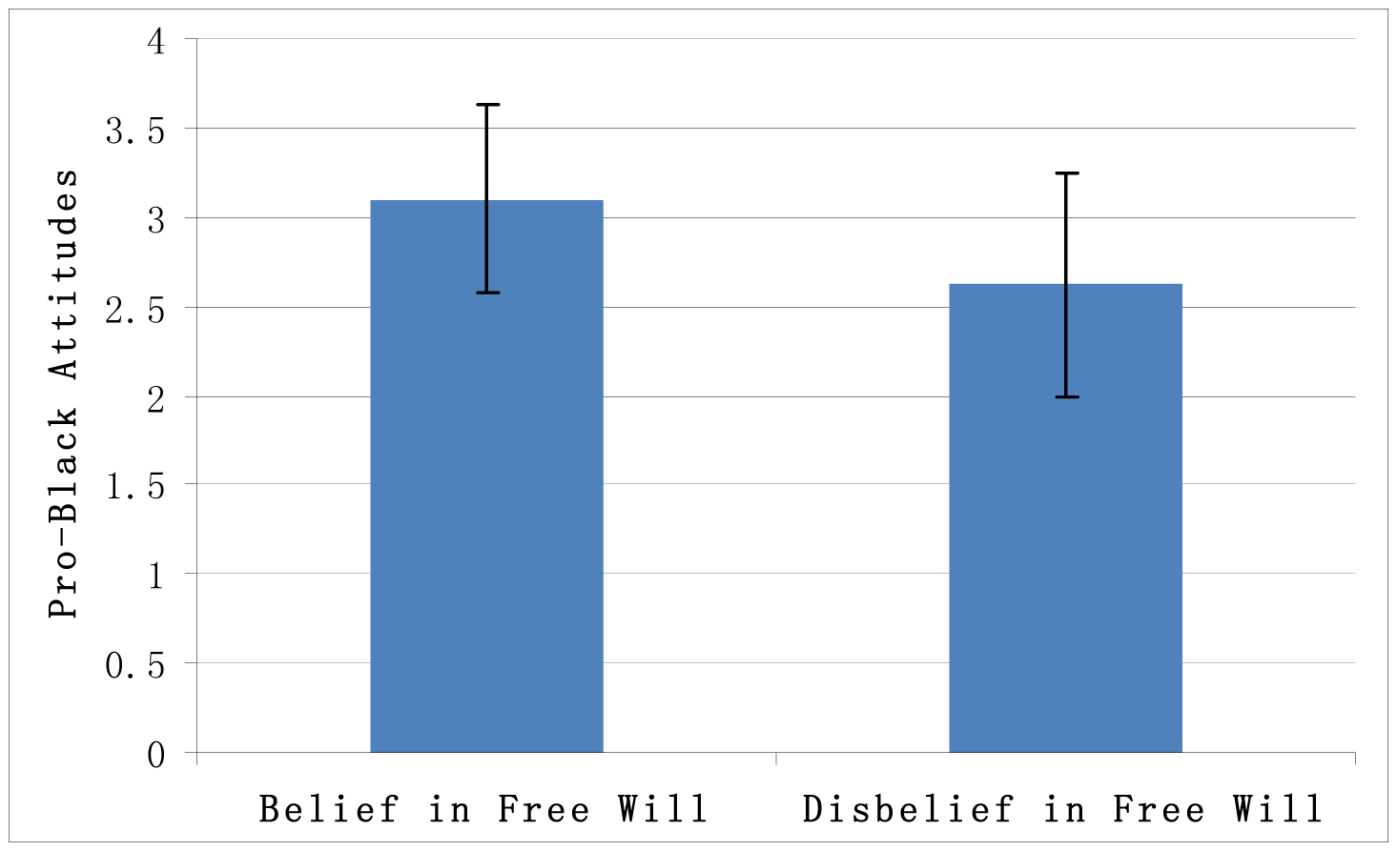
Pro-black attitudes in the belief in free will condition and the disbelief in free will condition.

To test the effect of belief in free will on prejudice against black people, we conducted an independent-samples t-test. The results revealed that, in the condition of belief in free will (*M* = 3.10, *SD* = 0.53), the participants expressed greater pro-black attitudes than did those in the condition of disbelief in free will (*M* = 2.62, *SD* = 0.63), *t*(61)  = –3.28, *p* = .002, *Cohen’s d* = 0.82 (Figure 3). Hence, Study 3 further confirmed hypothesis 2.

Notably, the results of Study 3 may have been confounded by the countries of origin of the subjects. The participants’ attitudes toward black people and their responses to the priming may have differed across countries, and this effect may also have accounted for the low reliability of the employed scale. However, due to the sample sizes from each country, we could not test the invariance of the results across counties. Future research studies employing larger sample sizes may directly test this hypothesis in each country.

## Discussion

This research reports the results of three studies that were designed to test our argument that belief in free will can predict and lead to reduced prejudice towards outgroups relative to disbelief in free will. We used different participant samples (Han Chinese and whites), explored the effects of belief in free will on prejudice against three outgroups (Tibetan Chinese, Uyghur Chinese, and blacks), and obtained consistent results. The Han Chinese with high levels of belief in free will were more likely to show less prejudice towards Tibetan Chinese. Compared with those who were primed to disbelieve in free will, the Han Chinese (whites) who were primed to believe in free will also exhibited less prejudice against Uyghur Chinese (blacks). The combination of these different samples and multiple methods in a single study has improved the generalizability of our findings.

Past studies have found that belief in free will provides a type of volitional willpower that motivates the self to exert socially desirable responses, such as increased pro-social behaviors and reduced aggression and cheating behaviors [10]–[11]. Despite the significance attached to the building of belief in free will on social behaviors, there has been a lack of empirical research that has exclusively investigated the association between the belief in free will and prejudice. Undoubtedly, socially desirable behaviors must include unprejudiced responses towards outgroups. The findings of the present research provide evidence that beliefs in free will lead to reduced ethnic/racial prejudice compared to disbeliefs in free will. Moreover, it is notable that the previous literature has established several pathways for reducing prejudice that can be categorized into interpersonal and intrapersonal approaches. Examples of the interpersonal approach include the findings that intergroup contact [29]–[30] and common ingroup identity [31] can ameliorate intergroup hostility. Examples of the intrapersonal approach include the findings that taking the perspective of the outgroup [32] and the implicit theory of personality [33] may promote intergroup amity. The present research contributes to the literature by adding a new perspective of the prejudice of the intrapersonal approach by indicating that one’s personal belief in free will is a possible antecedent of prejudice.

Moreover, as indicated in the literature, self-control may play a key mediating role in the effect of belief in free will on reducing prejudice. When people espouse reduced beliefs in free will, they may be reluctant to exert willpower to demonstrate their unprejudiced personal values (i.e., the internal motivation to control prejudice) or to satisfy the expectation of the social norm (i.e., the external motivation to control prejudice) [34]. Future research should directly test this mediating process.

In practice, the current research may also shed light on public crisis management. For example, the interracial disputes that occurred after Hurricane Katrina attracted a great deal of attention in the United States. After Hurricane Katrina, black people attributed the slow response of the government to racism, and whites believed that the black residents were themselves responsible for their misfortunes [35]. The present findings may shed light on this and related phenomena. People’s belief in free will can decrease within a certain period following a natural disaster [36]–[37]. Our results indicate that when people’s beliefs in free will are reduced, the expression of prejudice may exhibit corresponding increases. The increase in racial prejudice against the outgroup after Hurricane Katrina may due to changes in beliefs in free will. However, as natural disasters (e.g., hurricanes and earthquakes), epidemics of infectious disease (e.g., SARS and H5N1 bird flu), and social crises (e.g., terrorist attacks and economic crises) continue to occur, the identification of effective paths to avoid the occurrences of such intergroup conflicts is imperative. Based on the current research, a possible alternative solution to such conflicts might to advocate beliefs in free will through the public media during the recoveries from such catastrophes.

No research has ever been performed without limitations, and the present research is no exception. The present research is exploratory in the sense that it attempted to establish a link between belief in free will and prejudice. The theoretical premise that the present research adopted therefore remains a tentative proposition whose main function is to open up new avenues for research and to shed a different light on the fight against prejudice. However, the limited ambition of the present research cannot entirely excuse some of its problems. The most apparent limitation of Studies 2 and 3 are the absences of control conditions. The effect of belief in free will on prejudice was examined only in comparison with the condition of disbelief in free will. Thus, we cannot make the solid conclusion that belief in free will can act as an antidote to prejudice. There is a possible alternative explanation to our findings from Studies 2 and 3. Priming participants with disbelief in free will may have undermined their self-control, and they may have been more willing to self-report their prejudices relative to their counterparts who were exposed to the condition of belief in free will. The second limitation is that we used only three self-report measurements to assess ethnic/racial prejudices toward minorities. Although these three measurements differ in the content of the prejudices they measured (social interaction, affection, and positive attitude), behavioral measures that include approach and avoidance behaviors may overcome the pitfalls of our self-report measures (e.g., a single-item measure was used in Study 2) and improve the generalizability of our findings. Thirdly, the sample size of Study 2 was relatively small, and thus the findings should be interpreted with caution. In summary, future research should include a control condition and enlarge the sample size to confirm the current findings through the use of behavioral measurements.

## Supporting Information

Questionnaire S1 **Belief in free will scale.**
(DOC)

Questionnaire S2 **Social distance scale against Tibetan Chinese.**
(DOC)

Questionnaire S3 **Pro-black attitudes questionnaire.**
(DOC)

Priming materials S1 **12 statements in priming belief in free will and disbelief in free will.**
(DOC)

Priming materials S2 **Priming passages for belief in free will condition and disbelief in free will condition.**
(DOC)
