# Supplementary material for: The Effect of Belief in Free Will on Prejudice
Source: PLoS One. 2014 Mar 12;9(3):e91572. doi: 10.1371/journal.pone.0091572 (PMC3951431; doi:10.1371/journal.pone.0091572)
Supplement: Questionnaire S1 — Belief in free will scale. (DOC) [file pone.0091572.s001.doc]

### Questionnaire S1, Belief in free will scale

| 1 | People have complete control over the decision they make. |
| --- | --- |
| 2 | People must take full responsibility for any bad choices they make. |
| 3 | People can overcome any obstacles if they truly want to. |
| 4 | Criminals are totally responsible for the bad things they do. |
| 5 | People are always at fault for their bad behavior. |
| 6 | Strength of mind can always overcome the body’s desires. |
