# Supplementary material for: The Effect of Belief in Free Will on Prejudice
Source: PLoS One. 2014 Mar 12;9(3):e91572. doi: 10.1371/journal.pone.0091572 (PMC3951431; doi:10.1371/journal.pone.0091572)
Supplement: Questionnaire S2 — Social distance scale against Tibetan Chinese. (DOC) [file pone.0091572.s002.doc]

### Questionnaire S2, Social distance scale against Tibetan Chinese

| 1 | I do not mind living in the same community with Tibetans. |
| --- | --- |
| 2 | If my child were to be adopted by others, I would not want his/her step-parents to be Tibetans. |
| 3 | I do not mind if my neighbors are Tibetan. |
| 4 | I hope to know and make more Tibetan friends. (R) |
| 5 | I do not want Tibetans and Han Chinese to live together. |
