# Supplementary material for: The Effect of Belief in Free Will on Prejudice
Source: PLoS One. 2014 Mar 12;9(3):e91572. doi: 10.1371/journal.pone.0091572 (PMC3951431; doi:10.1371/journal.pone.0091572)
Supplement: Questionnaire S3 — Pro-black attitudes questionnaire. (DOC) [file pone.0091572.s003.doc]

### Questionnaire S5, Pro-black attitudes questionnaire

1 Black people do not have the same employment opportunities that whites do.

2 It's surprising that black people do as well as they do, considering all the obstacles they face.

3 Too many blacks still lose out on jobs and promotions because of their skin color.

4 Most blacks are no longer discriminated against.

5 The typical urban ghetto public school is not as good as it should be to provide equal opportunities for blacks.

6 This country would be better off if it were more willing to assimilate the good things in black culture.
