# Supplementary material for: The Effect of Belief in Free Will on Prejudice
Source: PLoS One. 2014 Mar 12;9(3):e91572. doi: 10.1371/journal.pone.0091572 (PMC3951431; doi:10.1371/journal.pone.0091572)
Supplement: Priming materials S1 — 12 statements in priming belief in free will and disbelief in free will. (DOC) [file pone.0091572.s004.doc]

### Priming materials S3, 12 statements in priming belief in free will and disbelief in free will

Priming of belief in free will

1 People have complete control over the decisions they make.

2 People must take full responsibility for any bad choices they make.

3 People can overcome any obstacles if they truly want to.

4 Criminals are totally responsible for the bad things they do.

5 People are always at fault for their bad behavior.

6 Strength of mind can always overcome the body’s desires.

Priming of disbelief in free will

1 The future has already been determined by fate.

2 No matter how hard you try, you can’t change your destiny.

3 Fate already has a plan for everyone.

4 Whatever will be, will be—there’s not much you can do about it.

5 Whether people like it or not, mysterious forces seem to move their lives.

6 Fate determines one’s success and failure.
