# Supplementary material for: The Effect of Belief in Free Will on Prejudice
Source: PLoS One. 2014 Mar 12;9(3):e91572. doi: 10.1371/journal.pone.0091572 (PMC3951431; doi:10.1371/journal.pone.0091572)
Supplement: Priming materials S2 — Priming passages for belief in free will condition and disbelief in free will condition. (DOC) [file pone.0091572.s005.doc]

### Priming materials S4, Priming passages for belief in free will condition and disbelief in free will condition

Priming of belief in free will

Usefulness of Volitional Control for Humankind

By Jonathan Inger           *Science* 13 February 2012   Vol. 311. no. 5766, p. 1341

DOI: 10.1126/time.311.5766.1341

Are people’s attitude and behaviors always controlled by volition? Can people’s brain truly dominate people’s reaction to various natural and social challenge? After conducting extensive field study, behavioral experiment, and neural cognitive study with functional MRI, the Cognition Research Centre at the University of Geneva concluded large numbers of human’s behaviors are indeed determined by our desire. A well-known cognitive psychologist, Professor George Levinger, the principle investigator of this project, also reviewed a large number of research findings from biology, psychology, archaeology, and cultural anthropology on people’s attitude and decision. The results suggested that, most of people’s attitudes and behaviors are controlled by themselves, like aggressive behaviors and altruism behaviors. He concluded that such controllability of our behaviors increase our survival probability in evolution…

Priming of disbelief in free will

Uselessness of Volitional Control for Humankind

By Jonathan Inger *Science* 13 February 2012 Vol. 311. no. 5766, p. 1341

DOI: 10.1126/time.311.5766.1341

Are people’s attitude and behaviors always controlled by volition? Can people’s brain truly dominate people’s reaction to various natural and social challenge? After conducting extensive field study, behavioral experiment, and neural cognitive study with functional MRI, the Cognition Research Centre at the University of Geneva concluded large numbers of human’s behaviors are indeed not determined by our desire. A well-known cognitive psychologist, Professor George Levinger, the principle investigator of this project, also reviewed a large number of research findings from biology, psychology, archaeology, and cultural anthropology on people’s attitude and decision. The results suggested that, in fact, plenty of people’s attitudes and behaviors are not controlled by themselves, like aggressive behaviors and altruism behaviors. He concluded that such uncontrollability of our behaviors increase our survival probability in evolution…
